# Detection of highly pathogenic avian influenza A(H5N1) virus, clade 2.3.4.4b, genotype B3.13 in placentas from aborted bovine fetuses

**DOI:** 10.1177/10406387261483207

**Published:** 2026-09-30

**Authors:** Melissa Macías-Rioseco, Sharif S. Aly, Javier Asin, Todd Cornish, Jennine Ochoa, Deniece R. Williams, Raúl A. Resendíz-Pozos, Robert B. Moeller, Wendi Jackson, Wagdy R. ElAshmawy, Beate Crossley, Francisco A. Uzal

**Affiliations:** California Animal Health and Food Safety Laboratory, Tulare, School of Veterinary Medicine, University of California–Davis, Davis, CA, USA; Department of Population Health and Reproduction, School of Veterinary Medicine, University of California–Davis, Davis, CA, USA; Veterinary Medicine Teaching and Research Center, School of Veterinary Medicine, University of California–Davis, Davis, CA, USA; San Bernardino branches, School of Veterinary Medicine, University of California–Davis, Davis, CA, USA; California Animal Health and Food Safety Laboratory, Tulare, School of Veterinary Medicine, University of California–Davis, Davis, CA, USA; California Animal Health and Food Safety Laboratory, Tulare, School of Veterinary Medicine, University of California–Davis, Davis, CA, USA; Veterinary Medicine Teaching and Research Center, School of Veterinary Medicine, University of California–Davis, Davis, CA, USA; California Animal Health and Food Safety Laboratory, Tulare, School of Veterinary Medicine, University of California–Davis, Davis, CA, USA; California Animal Health and Food Safety Laboratory, Tulare, School of Veterinary Medicine, University of California–Davis, Davis, CA, USA; Davis branches, School of Veterinary Medicine, University of California–Davis, Davis, CA, USA; Veterinary Medicine Teaching and Research Center, School of Veterinary Medicine, University of California–Davis, Davis, CA, USA; Department of Internal Medicine and Infectious Diseases, Faculty of Veterinary Medicine, Cairo University, Giza, Egypt; Davis branches, School of Veterinary Medicine, University of California–Davis, Davis, CA, USA; San Bernardino branches, School of Veterinary Medicine, University of California–Davis, Davis, CA, USA

**Keywords:** bovine abortion, dairy cattle, genotype B3.13, HPAI A(H5N1) clade 2.3.4.4b, influenza A virus

## Abstract

Infection by highly pathogenic avian influenza (HPAI) A(H5N1) virus (family *Orthomyxoviridae*, taxon species *Alphainfluenzavirus influenzae*), clade 2.3.4.4b, genotype B3.13, was first diagnosed in dairy cattle in the United States in March 2024. Here, we present a cluster of bovine abortions in whose placentas HPAI A(H5N1) was detected. Only placentas were submitted in all 5 cases received. Grossly, 3 placentas had multifocal edema; no significant gross abnormalities were observed in the other 2 placentas. Microscopically, one placenta had random areas of mineralization and another placenta had mild neutrophilic placentitis. Reverse-transcription quantitative real-time PCR for influenza A virus and avian influenza A virus subtype H5N1 were positive in all 5 placentas. RNAscope in-situ hybridization for matrix protein gene was positive in one placenta. Routine ancillary tests for bovine abortion diagnostic work-up did not detect any other abortifacient pathogens. We speculate that abortion was caused by the HPAI A(H5N1) virus.

In March 2024, the first diagnosis of highly pathogenic avian influenza (HPAI) A(H5N1) virus (family *Orthomyxoviridae*, taxon species *Alphainfluenzavirus influenzae*) infection of dairy cows was reported in Texas (https://www.aphis.usda.gov/livestock-poultry-disease/avian/avian-influenza/hpai-detections/hpai-confirmed-cases-livestock, accessed 2026 Apr 14). The virus was identified as H5N1, Eurasian lineage goose/Guangdong clade 2.3.4.4b, genotype B3.13. Most affected animals were multiparous cows in mid-to-late lactation. The dairy cattle affected by this virus had reduced feed consumption and an abrupt drop in milk production.^3^ Milk from these cows was discolored and thickened, resembling colostrum.^3,8^ Avian influenza A virus (**IAV**) has since been detected in milk by PCR, in the mammary tissue by immunohistochemistry (IHC), and in respiratory tissue, lymph nodes, heart, spleen, colon, liver, and kidney by in-situ hybridization.^3,4,12^

Here, we present a cluster of bovine abortions in the placentas of which HPAI A(H5N1) virus, clade 2.3.4.4b, genotype B3.13 was detected. A search performed on 2026 Jan 3 of PubMed, Google Scholar, One Search, and CAB abstracts did not yield any publications about HPAI A(H5N1) virus associated with abortion, placentitis, or infection of the placenta in bovine abortions.

In October 2024, a bulk-tank milk sample from a previously naive dairy farm located in Kern County, CA, USA, was positive for HPAI A(H5N1) virus by reverse-transcription quantitative real-time PCR (RT-qPCR). Twenty-five days later and within a 24-h period, a cluster of five ~23-mo-old Holstein heifers aborted in their last month of gestation. Approximately 24 h after the abortions, the heifers were examined clinically. Only dam 5 was clinically ill (sternal recumbency with decreased feed consumption). The placentas from the 5 heifers were gently pulled to exteriorize them as much as possible and cut close to the vulva. A new set of clean gloves was used for each heifer, and each placenta sample was placed in a clean, individual plastic bag. Vaginal swabs were taken from all 5 heifers. Colostrum samples were collected aseptically after disinfecting the teats with alcohol. Blood samples were collected from the 5 animals in tubes with no anticoagulants or other additives. All samples were transported on wet ice to the Tulare branch of the California Animal Health and Food Safety (CAHFS) laboratory the day of collection for a full diagnostic work-up. The fetuses were sent to rendering by the dairy farm and were therefore not available for examination or diagnostic work-up.

On gross examination, the placentas were all in a moderate state of postmortem decomposition. No gross lesions were observed in placentas from cases 1 and 5. Cases 2–4 had mild intercotyledonary edema. Samples of the chorioallantoic membrane, including cotyledons and intercotyledonary areas (cases 2–5), and amnion (case 1), were collected and fixed by immersion in 10% neutral-buffered formalin (pH 7.2) for ~24 h and processed routinely for the production of 4.0-µm-thick H&E-stained sections. Briefly, case 2 had multifocal mineralization in the cotyledonary portion (**Table 1**; **Fig. 1A**). In case 3, the trophoblasts were multifocally sloughed, and the chorionic membrane was covered by a moderate amount of cellular debris and small numbers of neutrophils (**Fig. 1B**). Moderate edema was observed within the intercotyledonary regions of case 4. No microscopic abnormalities were observed in the placentas of cases 1 and 5.

**Table 1.** Macroscopic and microscopic placental lesions in 5 bovine placentas positive for highly pathogenic influenza (HPAI) A(H5N1) virus, clade 2.3.4.4b, genotype B3.13, with corresponding placental RT-PCR results and serologic and RT-PCR findings from the associated dams.

Case	Sample	Macroscopic findings	Microscopic findings	PCR for IAV, Ct	PCR for IAV H5, Ct	PCR for H5 GsGD, Ct	IAV ELISA		IAV matrix real-time PCR		
							Milk (S:N)	Serum (S:N)	Milk	Vaginal swab	Ct
1	Amnion	Opaque membranes	Moderate autolysis	24.3	29.5	32.2	+ (0.27)	+ (0.15)	–	+	23.2
2	Chorioallantoic membranes with cotyledon	Intercotyledonary edema	Moderate multifocal mineralization	24.3	28.9	31.9	– (0.75)	+ (0.23)	–	+	22.9
3	Chorioallantoic membrane with cotyledon	Thickened, opaque membranes with edema	Sloughing of trophoblasts and mild-to-moderate neutrophilic placentitis	25.3	28.1	31.4	+ (0.24)	+ (0.17)	–	+	21.3
4	Chorioallantoic membrane with cotyledon	Intercotyledonary edema	Moderate edema	23.25	23.64	26.51	+ (0.07)	+ (0.20)	–	+	22.15
5	Chorioallantoic membrane with cotyledon	No gross lesions observed	Mild autolysis	22.12	24.83	27.55	+ (0.33)	– (0.74)	–	+	20.17

GsGD = Goose/Guangdong; IAV = influenza A virus; – = negative; + = positive; S:N = sample:negative ratio.

**Figure 1.**
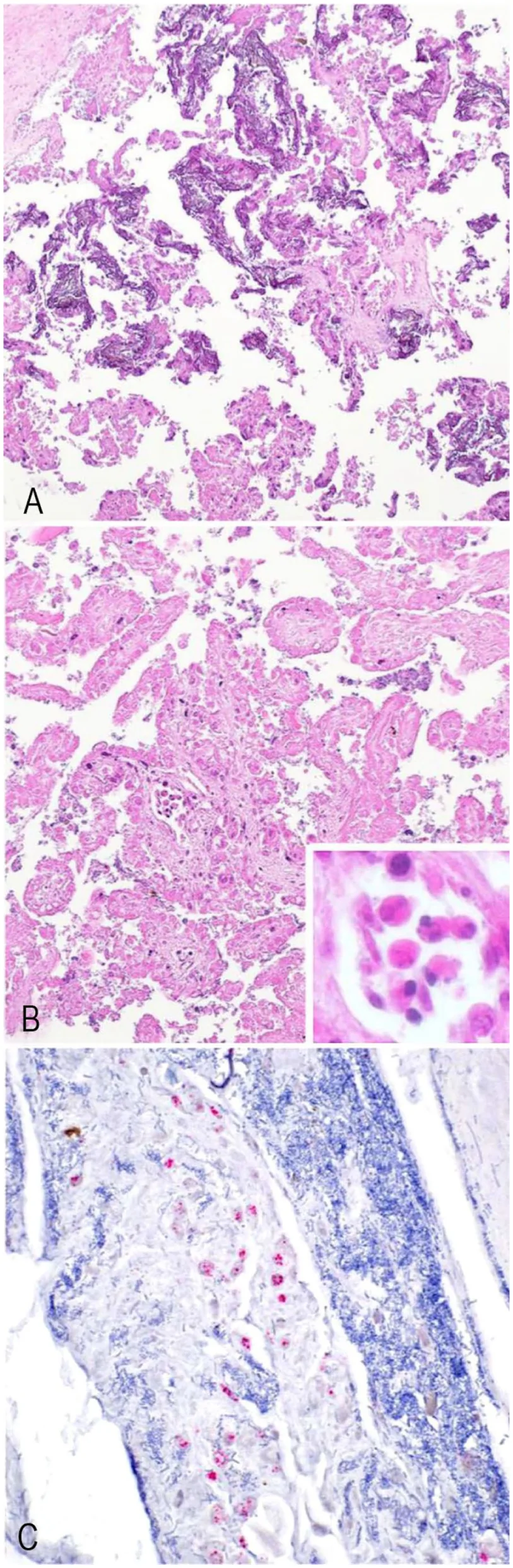
Microscopic findings of the HPAI A(H5N1) virus, clade 2.3.4.4b, genotype B3.13 PCR-positive placentas from dairy heifer abortions. **A.** Multifocal areas of mineralization in case 2. H&E. **B.** Trophoblasts have sloughed, and cotyledonary villi are covered with cellular debris and moderate numbers of neutrophils in case 3. Inset: higher magnification of sloughed trophoblasts. H&E. **C.** Colorimetric RNAscope in situ hybridization, with scattered labeling of trophoblasts in the allantois in case 2.

Selected sections of amnion from case 1 and chorioallantoic membrane from cases 2–5 were processed for IAV immunohistochemistry (IHC). Antigen retrieval was performed in a decloaking chamber, following quenching of the endogenous peroxidase with 3% hydrogen peroxide. Slides were incubated with mouse monoclonal antibodies targeting IAV nucleoprotein (C65331M; Meridian) for 1.25 h and later with an anti-mouse IgG antibody (12000; Vector). The slides were then washed and incubated in 3-amino-9-ethylcarbazole (AEC, RTU K3464; Dako) or Romulin AEC (RAEC810L; Biocare) reagent. Following the manufacturer’s instructions, an anti-species horseradish peroxidase–labeled polymer (Biocare) and a detection system with AEC (Thermo) were used. No immunoreactivity was detected on any of the sections examined. Appropriate negative (bovine placenta that tested negative for avian IAV by RT-qPCR) and positive (mammary tissue from an IAV RT-qPCR–positive cow) controls were included. Samples from the same blocks used for IHC were processed for RNAscope in-situ hybridization,^4,6,11^ with a probe targeting the matrix gene (cat. no. 1048221-C1; ACDBio), and evidence of viral RNA was observed in the allantois of case 2 (**Fig. 1C**).

Swabs collected aseptically from the chorioallantoic membrane (cases 2–5) and amnion (case 1) were inoculated onto blood and MacConkey agar and incubated at 37°C under aerobic conditions. All specimens were also inoculated into Campy FDA agar (Brucella agar with 7% lysed horse blood and PVANT antibiotics) and incubated at 37°C in an atmosphere of 5–10% carbon dioxide for 4 d.^5^ *Mannheimia granulomatis* was isolated from case 1, *Streptococcus uberis* was isolated from case 2, and no bacterial isolates were obtained from cases 3–5.

Additional swabs were collected aseptically from the chorioallantoic membrane (cases 2–5) and amnion (case 1) in transport medium (PrimeStore; ThermoFisher) for molecular testing. In preparation for whole-genome sequencing, the 8 influenza gene segments were amplified simultaneously in a single reaction using an RT-qPCR assay^14–16^ with the following universal primers: MBTuni-12 (0.1 µM), MBTuni-12.4 (0.1 µM), and MBTuni-13 (0.2 µM).^18^ A 25-µL reaction was used (Invitrogen Superscript III one-step RT-qPCR system, Platinum Taq DNA polymerase high fidelity; ThermoFisher), along with the following thermocycling reactions: 60 min at 42°C, 2 min at 94°C, then 5 cycles of 30 s at 94°C, 30 s at 44°C, 3 min at 68°C, followed by 31 cycles of 30 s at 94°C, 30 s at 57°C, 3 min at 68°C, and a final extension at 68°C for 7 min. For DNA library preparation, 500 ng of purified universal RT-qPCR amplicon from each sample was used as input for a library generated with the ligation sequencing kit SQK-LSK114 (Oxford Nanopore Technologies [ONT]).^7^ First, DNA was end-repaired using the NEBNext Ultra II end repair/dA tailing module (New England Biolabs), purified using AMPure XP beads (Beckman Coulter) in a ratio of 1:1 volume of beads per sample, and eluted in 30 µL of nuclease-free water. Sequencing adapters (AMX; ONT) were ligated to the DNA (Blunt/TA ligation master mix; New England Biolabs) by incubation at room temperature for 10 min. The adapter-ligated DNA library was purified using AMPure XP beads in a ratio of 1:2.5 volume of beads per sample, followed by 2 washes with S Fragment buffer (ONT) and elution in 7 µL of elution buffer (ONT). The library was loaded onto a Flongle flow cell (R10) on a GridION sequencer (ONT) and analyzed using MinKNOW software (v.25.05.14; ONT) for 24 h. Base-called FASTQ files containing “pass” reads (Q-score ≥10) were loaded into Geneious Prime (v.2022.1.1; Biomatters) and mapped to a reference bovine HPAI A(H5N1) virus genome using the MiniMap2 (v.2.24) plugin. Primer sequences were manually trimmed, and final consensus sequences with a minimum 100× depth of coverage across each entire segment were submitted to GenBank and assigned PV091373–PV091380. The IAV RT-qPCR, H5 Goose/Guangdong (GsGD) IAV RT-qPCR, and avian IAV H5 gene PCR assays were positive in all 5 cases. These placentas were RT-qPCR positive with Ct values of 22.1–25.3 (Table 1). RT-qPCR for IAV was conducted on milk and vaginal swab samples from all the heifers. All vaginal swabs were positive for IAV RT-qPCR, and all milk samples were negative on the same test (Table 1).

Serology for IAV was performed on milk and serum samples from the 5 dams (AI MultiS-Screen; Idexx). This assay is a blocking ELISA that detects antibodies to the IAV nucleoprotein in multiple animal species. Milk was considered positive if the sample:negative (S:N) ratio was <0.60; serum was considered positive if the S:N ratio was <0.40. Antibodies to IAV were not detected in the milk of dam 2; the rest of the dams were positive for IAV antibodies in milk. Dams 1–4 were positive for IAV antibodies in serum (Table 1).

Based on the detection of HPAI A(H5N1) virus, clade 2.3.4.4b, genotype B3.13 in the 5 placentas, coupled with the negative results for other abortifacient agents, a diagnosis of HPAI A(H5N1) virus infection was made, and we speculated that this virus was the cause of abortion. This first detection of HPAI A(H5N1) virus, clade 2.3.4.4b, genotype B3.13 in placentas from aborted dairy cattle is significant for understanding host adaptability, highlighting the potential for tissue affinity and interspecies transmission. The differences in antibody status between milk and serum in cases 2 and 5 may be interpreted as indicative of either an early or a very late stage of infection^2^; possibly the former, given that RT-qPCR from the milk of those animals was negative. However, a very late stage of infection is also plausible. The milk (colostrum) tested was RT-qPCR negative in the 5 dams; these first-calf heifers were not producing milk, and their mammae may not have been fully developed at the time of infection, making their milk RT-qPCR negative but antibody positive.

A limitation of our study was the lack of access to the fetuses for postmortem examination and the further serologic analysis of the dams. IHC was negative in all 5 placentas, which tested positive for IAV by RT-qPCR. The latter has higher sensitivity and can detect a low level of virus that might be missed in the specific tissue section used for IHC. Sample preservation could also have played a role given that the placentas were in a state of moderate postmortem decomposition. Abortion could have been caused by systemic illness or viremia. Hyperthermia can induce placental damage, and the release of inflammatory cytokines, such as IL1, TNF, and IL6, can trigger uterine contractions^1,17^ and predispose to abortion or fetal reabsorption in early pregnancies.^13^ However, because body temperatures of the dams were not recorded in our cases, this remains speculative. Likewise, infection of fetal tissues is a plausible scenario, which should be confirmed via examination of fetal tissues in cases similar to ours in the future.

The diagnosis of HPAI A(H5N1) virus infection in cattle and other species with lesions different from those traditionally described raises serious concerns about viral mutation and/or recombination, potentially affecting disease transmission dynamics and posing new risks to animal and public health. This virus has been detected in brain, lung, liver, stomach, and placenta from aborted alpaca fetuses^10^; that pasture-based farm included 1,100 chickens, 32 ducks, 18 alpacas, 13 yaks, 3 cats, 3 dogs, 1 llama, and 1 goat. Most animals had direct access to a pond filled by the stream bordering the property.^10^

Overall, HPAI A(H5N1) virus, clade 2.3.4.4b, genotype B3.13 in cattle is an operational challenge for livestock producers, potentially putting dairy owners, dairy workers, and veterinarians at risk of exposure from milk and from placentas and uterine fluids. The use of proper personal protective equipment is paramount to prevent zoonotic spread. Additionally, trade partners may impose restrictions on cattle or dairy products from affected regions, compounding financial losses and threatening market stability.

The detection of HPAI A(H5N1) virus, clade 2.3.4.4b, genotype B3.13 in placentas from dairy cattle with a history of abortion offers an additional opportunity to study the genetic changes that enable cross-species infection, contributing to knowledge about viral evolution and adaptation. The HPAI A(H5N1) virus was detected previously in South American sea lion fetuses, confirming transplacental HPAI A(H5N1) virus infection in a wild mammal, with viral antigens and RNA detected in the placenta and multiple fetal organs.^9^ Although the fetus had no gross lesions, IHC and RT-qPCR revealed widespread virus in the lungs, thymus, heart, and kidneys.^9^ Detection of HPAI A(H5N1) virus, clade 2.3.4.4b, genotype B3.13 in aborted placentas from dairy cattle suggests that this pathogen should be included in the differential diagnosis of abortion in dairy cattle experiencing HPAI A(H5N1) virus infection.

## References

[1] Arck PC , et al. Soluble receptors neutralizing TNF-α and IL-1 block stress-triggered murine abortion. Am J Reprod Immunol 1997;37:262–266. 10.1111/j.1600-0897.1997.tb00225.x 9127649

[2] Brown IH , et al. Detection of antibodies to influenza A virus in cattle in association with respiratory disease and reduced milk yield. Vet Rec 1998;143:637–638. 9881443

[3] Burrough ER , et al. Highly pathogenic avian influenza A(H5N1) clade 2.3.4.4b virus infection in domestic dairy cattle and cats, United States, 2024. Emerg Infect Dis 2024;30:1335–1343. 10.3201/eid3007.240508 PMC11210653 38683888

[4] Caserta LC , et al. Spillover of highly pathogenic avian influenza H5N1 virus to dairy cattle. Nature 2024;634:669–676. 10.1038/s41586-024-07849-4 PMC11485258 39053575

[5] Chaban B , et al. Isolation rates of *Campylobacter fetus* subsp *venerealis* from bovine preputial samples via passive filtration on nonselective medium versus selective medium, with and without transport medium. Am J Vet Res 2013;74:1066–1069. 10.2460/ajvr.74.8.1066 23879843

[6] Cronk BD , et al. Infection and tissue distribution of highly pathogenic avian influenza A type H5N1 (clade 2.3.4.4b) in red fox kits (*Vulpes vulpes*). Emerg Microbes Infect 2023;12: 2249554. 10.1080/22221751.2023.2249554 PMC10512766 37589241

[7] Crossley BM , et al. Nanopore sequencing as a rapid tool for identification and pathotyping of avian influenza A viruses. J Vet Diagn Invest 2021;33:253–260. 10.1177/1040638720984114 PMC7953088 33550926

[8] Eisfeld AJ , et al. Pathogenicity and transmissibility of bovine H5N1 influenza virus. Nature 2024;633:426–432. 10.1038/s41586-024-07766-6 PMC11390473 38977017

[9] Fiorito C , et al. Pathology of influenza A (H5N1) infection in pinnipeds reveals novel tissue tropism and vertical transmission. bioRxiv 2025. Preprint.

[10] Lehman KA , et al. Detection of highly pathogenic avian influenza A(H5N1) virus 2.3.4.4b in alpacas. J Vet Diagn Invest 2026. Advance online publication. doi:10.1177/10406387251414557. PMC12858382 41612781

[11] Pesavento PA , et al. In situ hybridization for localization of ovine herpesvirus 2 in formalin-fixed tissues. Vet Pathol 2018; 56:78–86. 10.1177/0300985818798085 30222071

[12] Righter DJ , et al. Detection of highly pathogenic avian influenza A(H5N1) clade 2.3.4.4b virus in cull dairy cows with underlying respiratory and systemic disease. J Vet Diagn Invest 2026. Advance online publication. doi:10.1177/10406387261417354. PMC12904801 41685536

[13] Rutigliano HM , et al. Increased expression of pro-inflammatory cytokines at the fetal–maternal interface in cloned bovine pregnancies. Am J Reprod Immunol 2022;87:e13520. 10.1111/aji.13520 PMC9285385 34974639

[14] Wise MG , et al. Development of a real-time RT-PCR for detection of Newcastle disease virus RNA. J Clin Microbiol 2004;42:329–338. 10.1128/JCM.42.1.329-338.2004 PMC321685 14715773

[15] Xing X , et al. Evolution and biological characterization of H5N1 influenza viruses bearing clade 2.3.2.1 hemagglutinin gene. Emerg Microbes Infect 2024;13:2284294. 10.1080/22221751.2023.2284294 PMC10769554 37966008

[16] Xing Z , et al. Inability of real-time RT-PCR to detect subtype H7 avian influenza viruses isolated from wild birds. J Clin Microbiol 2008;46:1844–1846. 10.1128/JCM.02426-07 PMC2395069 18322070

[17] Zenclussen AC , et al. Murine abortion is associated with enhanced interleukin-6 levels at the feto-maternal interface. Cytokine 2003;24:150-60. 10.1016/j.cyto.2003.08.002 14572793

[18] Zhou B Wentworth DE . Influenza A virus molecular virology techniques. Methods Mol Biol 2012;865:175–192. 10.1007/978-1-61779-621-0_11 22528160

